# Delayed hip arthroscopy for femoroacetabular impingement syndrome does not increase revision but does increase rates of chronic opiate use

**DOI:** 10.1016/j.jor.2024.02.046

**Published:** 2024-02-28

**Authors:** Kian Niknam, Ryan Freshman, Sergio E. Flores, Drew A. Lansdown, Stephanie E. Wong, Alan L. Zhang

**Affiliations:** Department of Orthopaedic Surgery, University of California - San Francisco, San Francisco, CA, USA

**Keywords:** Femoroacetabular impingement syndrome, Revision hip arthroscopy, Total hip arthroplasty, Post-operative opioid use

## Abstract

**Introduction:**

In recent years, the utilization of hip arthroscopy to treat femoroacetabular impingement syndrome (FAIS) has increased due to its low complication rates, positive impact on patient-reported outcomes (PROs), and association with faster rehabilitation. Despite this, there are high rates of revision and conversion to total hip arthroplasty (THA) in some of these patients. It is unclear whether time from initial FAIS diagnosis to surgery is a risk factor for poor outcomes. In this study, we examined the relationship between timing of hip arthroscopy for FAIS and rates of 2-year revision hip procedures, 2-year conversion to total hip arthroplasty (THA), post-operative medical complications, and opioid prescriptions.

**Methods:**

This is a retrospective cohort study utilizing the PearlDiver database. Current Procedural Terminology (CPT) and International Classification of Diseases (ICD) codes were used to identify patients who had surgery for FAIS with minimum 2 years follow-up available. Patients were stratified by 3-month intervals into 5 groups based on time from diagnosis of FAIS to hip arthroscopy. Multivariate logistic regression was performed to determine factors independently associated with continued opiate use and subsequent surgeries.

**Results:**

A total of 14,677 patients were included in the study. The 2-year rate of revision hip arthroscopy was 4.2%. As time from diagnosis to surgery increased, even in multivariate regression analysis, there was a higher risk of filling an opioid prescription 90 days after surgery (P < 0.001). Regression analysis demonstrated that timing of surgery was not associated with 2-year revision hip arthroscopy or conversion to THA. Age, sex, obesity, and tobacco use were significant predictors of revision hip arthroscopy and conversion to THA (p < 0.001).

**Conclusion:**

There is no significant difference between timing of surgery for FAIS and odds of revision or conversion to THA. Prolonged opiate use after hip arthroscopy was significantly higher as duration from initial FAIS diagnosis to surgery increased. Age, sex, obesity, and tobacco use are significant predictors for revision, conversion to THA, and continued opiate prescriptions.

## 1 Introduction

In recent years, the utilization of hip arthroscopy to treat femoroacetabular impingement syndrome (FAIS) has increased due to its low complication rates, positive impact on patient-reported outcomes (PROs), and association with faster rehabilitation.^1^ Despite largely positive results reported in the literature, rates of revision surgery after hip arthroscopy remain sobering, with 2-year revision hip arthroscopy surgery rates being as high as 15.1% and 2-year conversion to total hip arthroscopy (THA) being reported as high as 3.9%.^2^ Multiple demographic and pertinent risk factors, including patient age,^3^ pre-existing arthritic changes,^4^ opioid use,^5^ and higher pre-operative pain levels^4^ have been demonstrated to affect outcomes after hip arthroscopy for FAIS.

One factor that may also affect post-operative patient outcomes is timing of hip arthroscopy relative to initial diagnosis of FAIS. This concept has been explored within the ACL and rotator cuff literature, where delayed surgical management has led to higher rates of revision procedures and worse clinical outcomes.^6^, ^7^, ^8^ While several studies have investigated the effect of surgical timing and symptom duration on rates of revision hip arthroscopy and conversion to THA, their conclusions are limited by conflicting results and relatively small cohort sizes.^4^^,^^9^, ^10^, ^11^, ^12^, ^13^, ^14^ Additionally, despite the known relationship between opioid use and post-operative outcomes, these publications have not examined the effect that timing of surgery for FAIS has on post-operative pain symptoms and opioid use.^5^

The purpose of this study is to examine the relationship between timing of hip arthroscopy for FAIS and rates of 2-year revision hip procedures, 2-year conversion to THA, post-operative medical complications, and prolonged opioid prescriptions.

## 2 Methods

A retrospective cohort study of patients undergoing hip arthroscopy for FAIS from 2015 to 2021 was performed using the Mariner PearlDiver database (PearlDiver Technologies, Colorado Springs, CO). This database contains deidentified data from over 160 million patient lives from multiple insurance payer types. Current Procedural Terminology (CPT) and International Classification of Diseases (ICD)-10 codes (Appendix A) were used to identify patients undergoing hip arthroscopy for FAIS who had minimum of 2 years of follow-up. Patients were excluded from the analysis if they had codes for septic arthritis, acetabular fracture, femoral head fracture, loose bodies, and/or hip dislocation associated with their hip arthroscopy procedure (Appendix A).

Patients were then stratified into one of five cohorts based on their time from diagnosis of FAIS to surgery (less than 3 months, 3–6 months, 6–9 months, 9–12 months, greater than 12 months). Descriptive statistics, analysis of variance (ANOVA), and the chi-squared test were used to summarize the population's demographics and baseline characteristics. The identified cohorts were then compared with respect to rates of 2-year revision hip arthroscopy, 2-year conversion to THA, 90-day medical complications, and 90-day post-operative opioid prescriptions using the chi-squared test. Multivariate logistic regression was performed to assess and control for risk factors associated with revision hip arthroscopy. Factors put into these models included: timing to FAIS surgery, age, sex, Body Mass Index (BMI) > 30, Tobacco use, and Charlson Comorbidity Index (CCI). Multivariate regressions were also used to assess relationships associated with 2-year conversion to THA and chronic opiate use (defined as continued opiate prescriptions after 90 days from index surgery). A p-value of less than 0.05 was defined as statistically significant.

## 3 Results

A total of 14,677 patients met inclusion criteria for this study. Baseline patient demographics, including age, sex, BMI>30, tobacco use history and CCI were not significantly different between time-dependent cohorts. Pre-operative opioid use, however, was significant between time dependent cohorts with higher proportion of patients being prescribed opiates as timing to surgery increased (p < 0.001) (Table 1).

**Table 1:** Baseline patient demographics.

	<3 Months	3–6 Months	6–9 Months	9–12 Months	>12 Months	P-Value
Total No. of Patients (%)	12121 (82.6)	1476 (10.1)	429 (2.9)	151 (1.0)	500 (3.4)	–
Age, Mean (SD)	38.9 (14.0)	38.6 (13.7)	39.1 (13.4)	39.2 (14.6)	39.4 (13.5)	0.551
Male Gender, %	3523 (29.1)	416 (28.2)	119 (27.7)	46 (30.5)	133 (26.6)	0.686
Obesity, %	3463 (28.6)	417 (28.3)	146 (34.0)	42 (27.8)	131 (26.2)	0.101
Tobacco Use, %	3488 (28.8)	419 (28.4)	105 (24.5)	43 (28.5)	141 (28.2)	0.429
CCI, Mean (SD)	0.96 (1.24)	1.00 (1.36)	1.08 (1.35)	0.86 (1.23)	1.06 (1.21)	0.830
Pre-Operative Opioid Use, %	214 (1.8)	17 (1.2)	10 (2.3)	8 (5.3)	17 (3.4)	<0.001

Rates of 90-day readmission were not significantly different between time-dependent cohorts (p = 0.401). However, the rate of post-operative opioid prescriptions after 90 days from index surgery was significantly higher in patients with delayed surgery, ranging from 30.5% to 32.4% in cohorts who had surgery after 3 months of index FAIS diagnosis compared to 16.0% in the cohort consisting of patients who had surgery within 3 months of initial FAIS diagnosis (p < 0.001) (Table 2).

**Table 2:** Rates of 90-day post-operative readmission and medical complications, 90-day post-operative opioid prescriptions, and 2-year conversion to total hip arthroplasty and revision surgery.

	<3 Months	3–6 Months	6–9 Months	9–12 Months	>12 Months	P-Value
Readmission <90 days (%)	104 (0.9)	11 (0.8)	3 (0.7)	0 (0.0)	1 (0.2)	0.401
Urinary Tract Infection (UTI) (%)	228 (1.9)	35 (2.4)	7 (1.6)	1 (0.7)	11 (2.2)	0.500
Pneumonia (%)	57 (0.5)	14 (0.9)	3 (0.7)	0 (0.0)	5 (1.00)	0.069
Hematoma (%)	20 (0.2)	1 (0.1)	0 (0.0)	0 (0.0)	1 (0.2)	0.772
Superficial Wound Infection (%)	15 (0.1)	4 (0.3)	2 (0.5)	0 (0.0)	2 (0.4)	0.145
Acute Kidney Injury (%)	20 (0.2)	0 (0.0)	0 (0.0)	0 (0.0)	1 (0.2)	0.483
Deep Venous Thrombosis (%)	43 (0.4)	5 (0.3)	0 (0.0)	1 (0.7)	1 (0.2)	0.683
Pulmonary Embolism (%)	3 (0.02)	0 (0.0)	0 (0.0)	0 (0.0)	0 (0.0)	0.959
Cardiac Arrest (%)	1 (0.01)	0 (0.0)	0 (0.0)	0 (0.0)	1 (0.2)	0.010
Septic Arthritis (%)	5 (0.04)	0 (0.0)	0 (0.0)	0 (0.0)	0 (0.0)	0.901
Post-Operative Opioid Prescription- within 90 days from surgery, %	3939 (32.5)	466 (31.6)	135 (31.5)	46 (30.5)	159 (31.8)	0.911
Post-Operative Opioid Prescription- 90 days after surgery, %	1936 (16.0)	470 (31.8)	137 (31.9)	46 (30.5)	162 (32.4)	<0.001
Conversion to THA within 2 Years (%)	393 (3.2)	49 (3.3)	16 (3.7)	5 (3.3)	10 (2.0)	0.590
Revision Within 2 Years	540 (4.5)	50 (3.4)	11 (2.6)	6 (4.0)	14 (2.8)	0.048

In multivariate regression analyses, there was no significant difference in 2-year revision hip arthroscopy between time-dependent cohorts after controlling for age, sex, BMI>30, tobacco use, and CCI. Older age (OR: 0.97, 95%CI 0.97–0.98, p < 0.001), and male sex (OR: 0.67 95%CI:0.55–0.82) were significantly associated with lower odds of revision surgery, while BMI>30 (OR:1.31, 95%CI: 1.09–1.57, p = 0.003) and history of tobacco use (OR:1.24, 95%CI: 1.03–1.49, p = 0.022) were significantly associated with higher odds of revision hip arthroscopy (Table 3).

**Table 3:** Multivariate logistic regression for patients undergoing any arthroscopic revision procedure.

	Odds Ratio (OR)	95% Confidence Interval (95% CI)	P-Value
Age	0.97	0.97–0.98	<0.001
Male Gender	0.67	0.55–0.82	<0.001
Obesity	1.31	1.09–1.57	0.003
Tobacco Use	1.24	1.03–1.49	0.022
CCI	1.06	0.99–1.14	0.070

Time From Diagnosis to Surgery			
<3 Months	1.00 (Reference)	N/A (Reference)	N/A (Reference)
3–6 Months	0.74	0.55–1.00	0.051
6–9 Months	0.56	0.30–1.02	0.058
9–12 Months	0.90	0.39–2.05	0.798
>12 Months	0.63	0.37–1.08	0.093

Similarly, multivariate regression showed no significant differences in 2-year conversions to THA between time-dependent cohorts. Older age (OR:1.07, 95%CI: 1.06–1.08), BMI>30 (OR:2.11, 95%CI: 1.74–2.55, p < 0.001), and history of tobacco use (OR:1.68, 95%CI: 1.38–2.04, p < 0.001) were significantly associated with an increase in odds of 2-year conversion to THA. Male sex (OR:0.69, 95%CI:0.55–0.85) was significantly associated with lower odds of 2-year conversion to THA (Table 4).

**Table 4:** Multivariate logistic regression for patients undergoing conversion total hip arthroplasty.

	Odds Ratio (OR)	95% Confidence Interval (95% CI)	P-Value
Age	1.07	1.06–1.08	<0.001
Male Gender	0.69	0.55–0.85	0.001
Obesity	2.11	1.74–2.55	<0.001
Tobacco Use	1.68	1.38–2.04	<0.001
CCI	1.02	0.96–1.09	0.474

Time From Diagnosis to Surgery			
<3 Months	1.00 (Reference)	N/A (Reference)	N/A (Reference)
3–6 Months	1.08	0.78–1.45	0.641
6–9 Months	1.17	0.67–1.91	0.556
9–12 Months	0.95	0.33–2.17	0.916
>12 Months	0.63	0.31–1.13	0.153

Multivariate regression showed that time to surgery was a significant predictor of opiate prescriptions after 90 days from index FAIs surgery, with patients who had surgery at least 3 months after initial FAIS diagnosis having more than twice the odds of continued opiate prescriptions (p < 0.001) (Table 5). Older age (OR:1.01, 95%CI:1.06–1.08, p < 0.001), obesity (OR: 1.39, 1.27–1.53, p < 0.001), CCI (OR: 1.21, 95%CI: 1.17–1.25, p < 0.001),tobacco use (OR:2.16, 95%CI:1.97–2.36, p < 0.001), and pre-operative opiate use (OR:2.47, 95%CI:1.89–3.21, p < 0.001) were significantly associated with increased of odds of opiate prescriptions after 90 days from initial surgery. Male sex (OR:0.77, 95%CI:0.70–0.85, p < 0.001) was associated with lower odds of opiate prescriptions 90 days after index FAIS surgery (Table 5).

**Table 5:** Multivariate Logistic Regression Assessing opiate use after 90 days from initial surgery.

	Odds Ratio (OR)	95% Confidence Interval	P-value
Age	1.01	1.00–1.01	<0.001
Male Gender	0.77	0.70–0.85	<0.001
Obesity	1.39	1.27–1.53	<0.001
Tobacco Use	2.16	1.97–2.37	<0.001
CCI	1.21	1.17–1.25	<0.001
Pre-operative Opiate Use	2.47	1.89–3.21	<0.001

Time From Diagnosis to Surgery			
<3 Months	1.00 (Reference)	N/A (Reference)	N/A (Reference)
3–6 Months	2.65	2.34–3.01	<0.001
6–9 Months	2.62	2.11–3.26	<0.001
9–12 Months	2.47	1.71–3.56	<0.001
>12 Months	2.73	2.23–3.34	<0.001

## 4 Discussion

The primary finding from our study was that delayed surgery was not associated with higher rates of 2-year revision hip arthroscopy or conversion to THA. However, delaying surgery for greater than 3 months did associate with higher rates of prolonged opioid use (>3 months).

In our study, we found a 2-year revision hip arthroscopy and conversion to THA rate of 4.2% and 3.2%. This corresponds well with the current literature. Migliorini et al. completed a recent systematic review to assess rates in revision and conversion rates in patients undergoing hip arthroscopy and found the rates to be 5.3% (range:1.3%–50%) and 3.8% (2.9%–16.0%), respectively, which are comparable to our findings.^15^ We also found that there was no significant difference in odds of 2-year revision hip arthroscopy or conversion to THA with increasing time from initial FAIS diagnosis to surgery after controlling for various demographic and baseline characteristics. There are few studies examining the relationship between surgical timing and hip arthroscopy outcomes but their results are heterogenous and limited by smaller sample size. For example, Basques et al.^12^ and Aprato et al.^13^ found that patients with chronic symptoms for over 2 years and 3 years, respectively, had increased rates of revision surgery, while Kunze et al.^10^ found no such difference at 5-year follow-up in a single-surgeon retrospective study comparing patients with greater than 2 years and less than 2 years of hip symptoms. Additionally, Ruzbarksy et al. found that timing from FAIS symptom onset to surgery did not affect PROs in adolescent populations.^14^ Although symptom duration and time to surgery may be helpful in assessing disease acuity and progression, it does not necessarily correlate with severity of disease pathology or dysfunction.^16^

We also noted that there was no significant difference in timing to hip arthroscopy and 2- year conversion to THA. Studies assessing surgery timing in FAIs patients and THA are limited. Saadat et al. found that symptom duration greater than 1.5 years increased the risk of undergoing early THA, however, only one single-center study with a sample size of 227 reported associations between symptom duration and conversion to THA in this systematic review.^4^^,^^17^ Additionally, a recent single-center study found that symptom duration was not a significant predictor of 2-year conversion to THA.^18^ Our study is advantageous in that it utilizes data from multiple sites. However, further prospective studies are required to validate the lack of association between symptom duration and conversion to THA that was observed in this study.

In our multivariate regressions, we noted that older age was associated with lower odds of revision hip arthroscopy but increased odds of conversion to THA. This corresponds well with the current literature, as multiple studies have reported this association. Cevallos et al. reported that 33% of revision hip arthroscopies occurred in patients under the age of 30, while 64% of conversions to THA after initial hip arthroscopy occurred in patients 50 years and above .^19^ Additionally, other demographics and baseline characteristics, including female sex and obesity, were found to be associated with higher odds of 2-year revision hip arthroscopy and conversion to THA. This is also supported by current literature. In Kester et al.‘s retrospective analysis of 3957 patients, females sex was associated with higher odds of revision hip arthroscopy and conversion to THA (OR: 1.8, p < 0.001; and OR:1.6, p < 0.001, respectively).^20^ Bech et al. also reported higher odds of revision and THA in obese patients with obese patients, with an increase in odds by 4.7 times and 2.2 times, respectively.^21^

Lastly, we observed high rates of 90-day post-operative opioid prescriptions, as well as a higher risk of filling an opioid prescription as duration of hip symptoms increased in our study. Overall rates of post-operative opioid prescriptions after 90 days from surgery was 18.7%. This correlates well with previous studies, as Beck et al. reported that 18.2% of hip arthroscopy patients required more than 1 refill on their opiate prescription.^22^ Additionally, multiple studies have demonstrated that pre-operative opiate use predicts prolonged post-operative opiate use.^22^, ^23^, ^24^, ^25^, ^26^ This observation correlates well with our study, as pre-operative opiate use was significantly associated with opiate prescriptions after 90 days from index surgery. It is conceivable that the lack of association between surgery timing and revision hip arthroscopy and conversion to THA may be mediated through the prolonged utilization of opiates in patients who have surgery at least 3 months after initial FAIS diagnosis. It is possible that those who delay surgery for FAI may incur more damage to surrounding structures, including the labrum and articular cartilage, which in turn can affect duration of surgery and surgical outcomes and causes prolonged post-surgical pain that requires prolonged opiate use. A prospective causal mediation analysis is merited to further validate this association. Providers should consider these findings when consulting patients with regards to timing of surgery after initial FAIS diagnosis and potential opiate dependency.

## 5 Limitations

While there are multiple advantages to using a large administrative database, including a large sample size, and improved statistical power, our study was subject to a number of limitations. The validity of our results was dependent on the accuracy of ICD-10 and CPT coding within the database. Given limitations within the database, we could analyze the time from injury diagnosis to surgery but were unable to ascertain the exact duration of patient symptoms. We were also unable to account for delays associated with lack of patient access to care or insurance authorizations. Additionally, we did not have direct access to pre-operative physical exam and radiographic findings, intra-operative evaluations of labrum and cartilage pathology, and post-operative patient-reported outcomes or oral morphine equivalents consumed. Finally, our utilization of opiate prescriptions as a proxy to measure opiate use may not be accurate, as continued opiate prescriptions are not necessarily correlated with increased opiate use. Also, it is possible that patients were prescribed opiates for reasons other than sequelae from hip arthroscopy. We were unable to specify what the opiates were prescribed for in our study, so rates of prolonged post-operative opiate use are likely overestimated. However, it is hard to explain the large variability in opiate prescriptions after 90 days from index surgery between cohorts if timing to surgery had no impact on prolonged opiate prescriptions. This suggests that time to surgery may, in part, be associated with increased and chronic opiate use. A prospective causal mediation analysis to assess how opiate prescriptions may mediate interactions between surgery timing and subsequent surgeries is required to validate the results of this study.

## 6 Conclusion

There was no significant difference between timing of hip arthroscopy after initial FAIS diagnosis and revision or conversion to THA surgeries. However, patients with longer durations to surgery exhibited higher rates of prolonged post-operative prescriptions. Obesity, tobacco use, and female sex were all associated with increased risk of revision hip arthroscopy and conversion to THA. Providers should consider the results of this study when counseling patients on timing of hip arthroscopy after they have been diagnosed with FAIS and potential opiate dependence with increased symptom duration.

## Ethical statement

This study's data was generated using Pearldiver, an all-payor claims-based database. All data and analyses were conducted on an encrypted virtual desktop. All data is deidentified and not made directly available to the user of the platform assuring the data is secure.

## Funding

No Funding sources to disclose.

## Permission

This was a retrospective database study that used deidentified patient data. Guardian/patient permission not applicable.

## CRediT authorship contribution statement

**Kian Niknam:** Conceptualization, Investigation, Methodology, Roles/. **Ryan Freshman:** Conceptualization, Investigation, Methodology, Roles/. **Sergio E. Flores:** Conceptualization, Investigation, Methodology, Roles/. **Drew A. Lansdown:** Conceptualization, Investigation, Methodology, Roles/. **Stephanie E. Wong:** Conceptualization, Investigation, Methodology, Roles/. **Alan L. Zhang:** Conceptualization, Investigation, Methodology, Roles/.

## Declaration of competing interest

Alan Zhang: Consultant for Stryker and Depuy-Mitek.

Drew Lansdown: Consultant for Vericel inc. and AlloSource.

The authors declare that they have no known competing financial interests or personal relationships that could have appeared to influence the work reported in this paper.

## Figures and Tables

**Table A:** Diagnostic and Procedural Codes for Patient, Risk Factor, and Outcome Identification

Concept	Code
Hip Arthroscopy (CPT)	
	29914, 29916, 29862, 29915, 29863, 29861, 29860
Femoroacetabular Impingement Syndrome (ICD-10)	
	M25851, M24151, M24851, S73191A, M25852, M24152, M24852, S73192A
Total Hip Arthroplasty	
	ICD-10-P-0SR9049, ICD-10-P-0SR904Z, ICD-10-P-0SR904A, ICD-10-P-0SR9039, ICD-10-P-0SR903Z, ICD-10-P-0SR903A, ICD-10-P-0SR9029, ICD-10-P-0SR902Z, ICD-10-P-0SR902A, ICD-10-P-0SR9019, ICD-10-P-0SR901Z, ICD-10-P-0SR901A, ICD-10-P-0SR9069, ICD-10-P-0SR906Z, ICD-10-P-0SR906A, ICD-10-P-0SR90J9, ICD-10-P-0SR90JZ, ICD-10-P-0SR90JA, ICD-10-P-0SRB07Z, ICD-10-P-0SRB049, ICD-10-P-0SRB04Z, ICD-10-P-0SRB04A, ICD-10-P-0SRB039, ICD-10-P-0SRB03Z, ICD-10-P-0SRB03A, ICD-10-P-0SRB029, ICD-10-P-0SRB02Z, ICD-10-P-0SRB02A, ICD-10-P-0SRB019, ICD-10-P-0SRB01Z, ICD-10-P-0SRB01A, ICD-10-P-0SRB0KZ, ICD-10-P-0SRB069, ICD-10-P-0SRB06Z, ICD-10-P-0SRB06A, ICD-10-P-0SRB0J9, ICD-10-P-0SRB0JZ, ICD-10-P-0SRB0JA
Hip Fracture (ICD-10)	
	S72041A, S72041C, S72041B, S72041S, S72041G, S72041K, S72041D, S72001A, S72001C, S72001B, S72001S, S72001G, S72001P, S72001K, S72001D, S72001J, S72001R, S72001 N, S72001F, S72001H, S72001Q, S72001 M, S72001E, S72044A, S72044B, S72044S, S72044G, S72044D, S72091A, S72091C, S72091B, S72091S, S72091G, S72091P, S72091K, S72091D, S72091 M, S72141A, S72141B, S72141D, S72141E, S72141F, S72141G, S72141J, S72141K, S72141 M, S72141P, S72141S, S72144A, S72144D, S72144F, S72144G, S72144H, S72144K, S72144 M, S72144P, S72144S, S72002A, S72002B, S72002C, S72002D, S72002E, S72002F, S72002G, S72002H, S72002J, S72002K, S72002 M, S72002P, S72002R, S72002S, S72042A, S72042B, S72042C, S72042D, S72042F, S72042G, S72042K, S72042 N, S72042P, S72042R, S72042S, S72045A, S72045D, S72045G, S72045K, S72045S, S72092A, S72092B, S72092C, S72092D, S72092E, S72092G, S72092H, S72092K, S72092 M, S72092 N, S72092P, S72092S, S72142A, S72142B, S72142C, S72142D, S72142E, S72142F, S72142G, S72142J, S72142K, S72142P, S72142S, S72145A, S72145B, S72145D, S72145E, S72145F, S72145G, S72145K, S72145R, S72145S
Septic Arthritis (ICD-9)	
	71105, 71195
Septic Arthritis (ICD-10)	M00059, M00159, M00259, M00859, M00051, M00052, M00251, M00252, M01X51, M01X52, M01X59, M0089
Superficial Infection (ICD-9)	
	99883, 99832, 99859, 99851
Superficial Infection (ICD-10)	
	T8131XA, T8131XD, T8131XS, T8141XA, T8141XS, T8141XD
Exclusion criteria-femoral neck fracture (ICD-9)	
	8208, 82100, 82021, 82009, 82020, 82000, 82022, 73314, 82003, 82002, 8209, 82001, 82010, 82011, 82012, 82013, 82019, 82030, 82031, 82032, 9053
Exclusion criteria-femoral neck fracture (ICD-10)	
	S72001A, S72001B, S72001C, S72001D, S72001E, S72001F, S72001G, S72001H, S72001J, S72001K, S72001M, S72001N, S72001P, S72001Q, S72001R, S72001S, S72002A, S72002B, S72002C, S72002D, S72002E, S72002F, S72002G, S72002H, S72002J, S72002K, S72002M, S72002P, S72002R, S72002S, S72009A, S72009B, S72009C, S72009D, S72009E, S72009G, S72009K, S72009N, S72009P, S72009R, S72009S, S72041A, S72041B, S72041C, S72041D, S72041G, S72041K, S72041S, S72042A, S72042B, S72042C, S72042D, S72042F, S72042G, S72042K, S72042N, S72042P, S72042R, S72042S, S72043A, S72043B, S72043D, S72043G, S72043K, S72043S, S72044A, S72044B, S72044D, S72044G, S72044S, S72045A, S72045D, S72045G, S72045K, S72045S, S72046A, S72046D, S72091A, S72091B, S72091C, S72091D, S72091G, S72091K, S72091M, S72091P, S72091S, S72092A, S72092B, S72092C, S72092D, S72092E, S72092G, S72092H, S72092K, S72092M, S72092N, S72092P, S72092S, S72099A, S72099B, S72099D, S72099F, S72099K, S72099S
Exclusion criteria-arthroplasty	
	CPT-27299, ICD-9-P-8151, ICD-9-P-0070, ICD-9-P-8152, ICD-9-P-0073, ICD-9-P-0072, ICD-9-P-0071, ICD-9-P-8005, ICD-9-P-8153, ICD-10-P-0SR904A, ICD-10-P-0SRB04A, ICD-10-P-0SR904Z, ICD-10-P-0SR902A, ICD-10-P-0SRB04Z, ICD-10-P-0SRB02A, ICD-10-P-0SR90JZ, ICD-10-P-0SR90JA, ICD-10-P-0SRB0JZ, ICD-10-P-0SRB0JA, ICD-10-P-0SR902Z, ICD-10-P-0SRB02Z, ICD-10-P-0SR903A, ICD-10-P-0SRB03A, ICD-10-P-0SR903Z, ICD-10-P-0SRB03Z, ICD-10-P-0SR901A, ICD-10-P-0SRR0JZ, ICD-10-P-0SRB01A, ICD-10-P-0SRR0JA, ICD-10-P-0SR9049, ICD-10-P-0SR90J9, ICD-10-P-0SRS0JA, ICD-10-P-0SRS0J9, ICD-10-P-0SRS0JZ, ICD-10-P-0SRR01A, ICD-10-P-0SR9029, ICD-10-P-0SRB0J9, ICD-10-P-0SR901Z, ICD-10-P-0SRB029, ICD-10-P-0SRS01A, ICD-10-P-0SRR0J9, ICD-10-P-0SRB049, ICD-10-P-0SRB06A, ICD-10-P-0SR906A, ICD-10-P-0SRR03A, ICD-10-P-0SRB01Z, ICD-10-P-0SRS03A, ICD-10-P-0SRR03Z, ICD-10-P-0SRS01Z, ICD-10-P-0SRR01Z, ICD-10-P-0SRS03Z, ICD-10-P-0SRR019, ICD-10-P-0SR906Z, ICD-10-P-0SRS019, ICD-10-P-0SR9019, ICD-10-P-0SRB06Z, ICD-10-P-0SRB039, ICD-10-P-0SR9039, ICD-10-P-0SRE0JZ, ICD-10-P-0SRA0JZ, ICD-10-P-0SRB019, ICD-10-P-0SRS039, ICD-10-P-0SR90EZ, ICD-10-P-0SRA00A, ICD-10-P-0SRR039, ICD-10-P-0SRA01A, ICD-10-P-0SRB0EZ, ICD-10-P-0SRE00A, ICD-10-P-0SRE00Z, ICD-10-P-0SRE0JA, ICD-10-P-0SRA01Z, ICD-10-P-0SRE01A, ICD-10-P-0SR9069, ICD-10-P-0SRA009, ICD-10-P-0SRA00Z, ICD-10-P-0SRA019, ICD-10-P-0SRA03A, ICD-10-P-0SRA03Z, ICD-10-P-0SRA0J9, ICD-10-P-0SRA0JA, ICD-10-P-0SRB069, ICD-10-P-0SRB07Z, ICD-10-P-0SRB0KZ, ICD-10-P-0SRE009, ICD-10-P-0SRE019, ICD-10-P-0SRE01Z, ICD-10-P-0SRE039, ICD-10-P-0SRE03A, ICD-10-P-0SRE03Z, ICD-10-P-0SRE0J9, ICD-10-P-0SRR07Z, ICD-10-P-0SRS07Z
Exclusion criteria- Femur ORIF	
	CPT-27236, CPT-27235, CPT-27187, CPT-27161, CPT-27170, CPT-27230, CPT-27232, ICD-9-P-7855, ICD-9-P-7915, ICD-9-P-7935, ICD-10-P-0QH604Z, ICD-10-P-0QH606Z, ICD-10-P-0QH634Z, ICD-10-P-0QH635Z, ICD-10-P-0QH636Z, ICD-10-P-0QH63CZ, ICD-10-P-0QH646Z, ICD-10-P-0QH704Z, ICD-10-P-0QH705Z, ICD-10-P-0QH706Z, ICD-10-P-0QH734Z, ICD-10-P-0QH735Z, ICD-10-P-0QH736Z, ICD-10-P-0QH744Z, ICD-10-P-0QP604Z, ICD-10-P-0QP605Z, ICD-10-P-0QP634Z, ICD-10-P-0QP704Z, ICD-10-P-0QP705Z, ICD-10-P-0QP734Z, ICD-10-P-0QP744Z, ICD-10-P-0QS604Z, ICD-10-P-0QS605Z, ICD-10-P-0QS606Z, ICD-10-P-0QS634Z, ICD-10-P-0QS635Z, ICD-10-P-0QS636Z, ICD-10-P-0QS63BZ, ICD-10-P-0QS646Z, ICD-10-P-0QS704Z, ICD-10-P-0QS706Z, ICD-10-P-0QS734Z, ICD-10-P-0QS735Z, ICD-10-P-0QS736Z, ICD-10-P-0QS744Z, ICD-10-P-0QS746Z, ICD-10-P-0QW604Z, ICD-10-P-0QW704Z, ICD-10-P-0QW705Z
Opiate use	
	USC-02211, USC-02212, USC-02214, USC-02221, USC-02222, USC-02231, USC-02232</monospace>
	USC-02211, USC-02212, USC-02214, USC-02221, USC-02222, USC-02231, USC-02232</monospace>
	2780, 27800, 27801, 27802, 27803,
Obesity (ICD-10)	
	E660:E669
Tobacco use	
	ICD-9-D-3051, ICD-9-D-V1582, F17220, F17221, ICD-10-D-F17223, F17228, F17229, F17290, F17291, F17293, F17298, F17299, Z720
